# Chronic Subthalamic Nucleus Stimulation in Parkinson's Disease: Optimal Frequency for Gait Depends on Stimulation Site and Axial Symptoms

**DOI:** 10.3389/fneur.2019.00029

**Published:** 2019-02-08

**Authors:** Irene Di Giulio, Eirini Kalliolia, Dejan Georgiev, Amy L. Peters, Daniel C. Voyce, Harith Akram, Thomas Foltynie, Patricia Limousin, Brian L. Day

**Affiliations:** ^1^Department of Clinical and Movement Neurosciences, Queen Square Institute of Neurology, University College London, London, United Kingdom; ^2^Centre for Human and Applied Physiological Sciences, School of Basic and Medical Biosciences, King's College London, London, United Kingdom; ^3^St. Luke's Hospital Thessaloniki, Thessaloniki, Greece; ^4^Department of Neurology, University Medical Centre, Ljubljana, Slovenia; ^5^Department of Physical Medicine and Rehabilitation, University of Colorado, Anschutz Medical Campus, Aurora, CO, United States

**Keywords:** parkinson's disease, gait, deep brain stimulation, subthalamic nucleus, axial symptoms

## Abstract

Axial symptoms emerge in a significant proportion of patients with Parkinson's disease (PD) within 5 years of deep brain stimulation (STN-DBS). Lowering the stimulation frequency may reduce these symptoms. The objectives of the current study were to establish the relationship between gait performance and STN-DBS frequency in chronically stimulated patients with PD, and to identify factors underlying variability in this relationship. Twenty-four patients treated chronically with STN-DBS (>4 years) were studied off-medication. The effect of stimulation frequency (40–140 Hz, 20 Hz-steps, constant energy) on gait was assessed in 6 sessions spread over 1 day. Half of the trials/session involved walking through a narrow doorway. The influence of stimulation voltage was investigated separately in 10 patients. Gait was measured using 3D motion capture and axial symptoms severity was assessed clinically. A novel statistical method established the optimal frequency(ies) for each patient by operating on frequency-tuning curves for multiple gait parameters. Narrowly-tuned optimal frequencies (20 Hz bandwidth) were found in 79% of patients. Frequency change produced a larger effect on gait performance than voltage change. Optimal frequency varied between patients (between 60 and 140 Hz). Contact site in the right STN and severity of axial symptoms were independent predictors of optimal frequency (*P* = 0.009), with lower frequencies associated with more dorsal contacts and worse axial symptoms. We conclude that gait performance is sensitive to small changes in STN-DBS frequency. The optimal frequency varies considerably between patients and is associated with electrode contact site and severity of axial symptoms. Between-subject variability of optimal frequency may stem from variable pathology outside the basal ganglia.

## Introduction

Axial symptoms, consisting of gait, balance and speech problems, are a serious and disabling feature of Parkinson's disease (PD). These symptoms can emerge in a significant proportion of patients (20–80%) who have been receiving deep brain stimulation of the subthalamic nucleus (STN-DBS) for up to 5 years (1–10). There is some evidence that the symptoms might be better controlled using low stimulation frequencies of 60–80 Hz rather than the higher frequencies of around 130 Hz that typically are implemented soon after implantation (11–14). However, not all studies have shown this effect of lowering the stimulation frequency (15, 16), suggesting there might be substantial heterogeneity within the PD population. The aim of this study was to measure the response to change in DBS stimulation parameters in individual patients with PD. We focused on changes in stimulation frequency, but undertook an additional experiment to investigate the relative effects of the stimulation voltage change that inevitably accompany frequency change when using a constant-energy stimulation protocol.

It is not understood why axial symptoms emerge in only some patients receiving STN-DBS, and why the axial symptoms in those patients might respond variably to stimulation frequency. To shed light on this we have identified, for the first time, the stimulation-frequency tuning curves and their confidence intervals for one aspect of motor behavior in individual PD patients who had been chronically stimulated for more than 4 years. The behavior we have chosen is gait, since gait difficulties represent an important component of the axial symptom complex. By ascertaining the frequency-tuning characteristics related to gait performance for individuals, we have been able to establish the variability of tuning within a chronically stimulated PD cohort and to identify some of the factors underlying this variability. This has enabled us to speculate on the potential role of lesions outside the basal ganglia on the emergence of axial symptoms.

## Materials and Methods

### Participants

Twenty-four PD patients (8 female) were recruited from the National Hospital for Neurology and Neurosurgery. Patients were eligible for the study if they had been implanted with STN-DBS 4 years or more before the test date and they could walk independently indoors. Patients with a known cognitive deficit were not recruited. Patients' clinical details and stimulation sites are reported in Table 1. Participants gave written, informed consent to the procedures, which conformed to the Declaration of Helsinki and were approved by the University College London Hospital NHS Trust ethics committee (13/LO/0379).

**Table 1:** Patient demographics and clinical details.

													Total UPDRS III post-surgery follow-up^*^		
**patient ID**	**Height (m)**	**Weight (Kg)**	**Age range (y)**	**disease duration (y)**	**Time since surgery (y)**	**Total LEDD (mg/day)**	**Hoehn and Yahr**	**Comorbidity**	**Falls**	**Assistive device**	**L STN electrode region**	**R STN electrode region**	**OFF- meds OFF- stim**	**OFF-meds ON- stim**	**ON-meds ON- stim**
OP01	1.63	73	70–75	17	10	200	3	No	Yes	No	c,s	p,s	84	13	13
OP02	1.74	87	66–70	19	5	932	3	Lower back pain, sciatica	Yes	Stick	i	i	22	11	7
OP03	1.64	69.7	60–65	18	9	500	3	Hip pain (no replacement)	Yes	No	c	c	47	19	12
OP04	1.79	108	56–60	13	8	1,969	3	No	Yes	Stick	i	p	38	25	7
OP05	1.73	77.5	60–65	12	4	599	2.5	No	No	No	p	s	32	18	10
OP06	1.75	92	70–75	12	6	354	2.5	No	Yes	Walker/ stick	i	p		21	10
OP07	1.73	84	66–70	28	13	540	3	No	Yes	No				19	8
OP08	1.59	86	66–70	19	6	560	2.5	High blood pressure	No	Stick	p,s	c	36	24	21
OP09	1.61	96	56–60	19	7	1,230	3	Lower back pain	Yes	Stick	s,a	c	67	56	11
OP10	1.72	83.5	60–65	13	8	988	2.5	No	No	No	s	s	43	10	5
OP11	1.88	92	46–50	13	7	1,115	3	No	Yes	No	s	c	51	21	10
OP12	1.7	99.5	60–65	11	4	550	2.5	No	Yes	Scooter, 2 sticks	i	i	46	27	15
OP13	1.61	70	60–65	18	7	254	2.5	No	No	No	i	a	53	17	7
OP14	1.77	93	56–60	19	5	754	3	No	Yes	No	s	c	52	30	15
OP15	1.6	76.5	56–60	14	5	1,650	2.5	No	Yes	No	p	p	30	22	10
OP16	1.59	72	70–75	28	10	1,500	3	No	Yes	Stick	a	c	37	13	5
OP17	1.64	73.6	50–55	13	8	700	2.5	No	No	No	p	i,c	83	42	18
OP18	1.64	95	60–65	18	10	975	2.5	Diabetes	No	Stick	c,c	i	48	20	18
OP19	1.5	74	60–65	20	11	720	3	Cervical spine surgery before DBS	Yes	Stick	c	s,s	41	16	7
OP20	1.66	79	56–60	15	9	500	3	Diabetes	Yes	Stick	a	i	36	16	13
OP21	1.8	100	50–55	13	6	0	3	No	No	Scooter outdoors	s	s	27	21	
OP22	1.6	71	50–55	10	4	72	3	No	Yes	No	c	i	68	44	25
OP23	1.67	82	50–55	12	4	700	2.5–3	No	No	No	p	i	52	24	11
OP24	1.65	90	60–65	20	5	600	3	Knee osteoarthritis, right knee surgery, lumbar back pain, sciatica, diabetes	Yes	Stick	p	p	40	19	17

*Active electrode regions: a, anterior medial; c, central; p, posterior lateral; s, superior; i, inferior. Two letters indicate bipolar stimulation with the site of each active electrode*.

* *Recorded at 1 year after surgery except for OP03 (3 y), OP06 (0.5 y), OP07 (7 y), OP22 (0.67 y), OP24 (2 y)*.

### Experimental Procedure

Patients visited the laboratory after at least 12 h of medication (levodopa + dopamine agonist) wash-out. The STN-DBS parameters were set, with patients blinded to the setting, and a 30-min “stabilization” time was observed before each test session, which lasted 20 min. A visit included tests of gait function and upper-limb function (not reported here), and a clinical assessment of axial symptoms using a reduced version of the UPDRS-III test (rising from a chair, posture, gait and pull test).

The gait protocol consisted of walking 8 m per trial for 16 trials at each STN-DBS setting. Some patients completed fewer trials for some settings, but never less than 8 trials, which was deemed adequate to be included in the analysis (patient OP18 completed 12 trials at 120 Hz; 8 trials were completed at 40 Hz for patients OP04, OP13, and OP22; at 60 Hz for patients OP08, OP21, and OP22; at 80 Hz for patient OP22; at 140 Hz for patients OP08, OP16, and OP18). At the beginning of half the trials selected randomly, a mock doorway was placed halfway along the walkway. The doorway consisted of two motor-driven, 300 mm-wide, floor-to-ceiling panels separated by a gap set to the patient's shoulder width. An auditory tone instructed patients to start walking at their preferred pace and manner. Gait performance was measured from infra-red markers placed bilaterally on the second metatarsal head and the heel. Marker positions were collected by a 6-unit motion capture system (CODA CX1; Charnwood Dynamics, Rothley, UK) at 100 Hz.

#### Experiment 1

Six experimental sessions were attempted during a single visit to study the effect of stimulation frequency. In each session STN-DBS frequency was changed in a random order. The range tested was 40–140 Hz with 20 Hz-steps, adjusting voltage stimulation in order to maintain the stimulation energy constant, according to the formula,

$$\begin{array}{l} Energy\left(TEED\right)=\frac{voltage^{2}\times pulse\;width\times frequency}{impedance} \end{array}$$

At the end of this experiment, the patients returned to their normal medication regime and DBS settings.

#### Experiment 2

In experiment 1 each change in stimulation frequency was accompanied by a voltage change to maintain constant stimulation energy. We therefore undertook an additional experiment to ascertain which of these two stimulation parameters had the greater effect on gait performance. The relative influence of frequency and voltage change was studied in a subset of 12 patients in a separate visit (median 338 days after experiment 1; range 2–539 days). The gait protocol was performed using four stimulation settings in random order (four sessions), combining two voltages at each of two stimulation frequencies (80 and 120 Hz). These stimulation frequencies were chosen because they represent typical low and high frequencies used clinically and they were best tolerated by the patients during experiment 1. The voltage levels were determined using the constant energy criterion for the high-frequency/low-voltage and low-frequency/high-voltage combinations. Because voltage was determined by frequency and constant energy, voltage levels were different across patients. The mean voltage difference between low and high setting was 0.82 V. Two patients completed only 3 sessions and were excluded from the analysis.

### Measurements

Stepping events were determined for each trial using custom scripts in Matlab (Mathworks, Natik, US). Local minima were identified on the antero-posterior heel and toe acceleration traces and classified as heel strikes and toe offs. These were visually inspected together with the markers' vertical positions, and manually adjusted if needed, via an interactive program (17, 18). These events were used to calculate gait performance variables, including stride length (distance in heel antero-posterior position between heel strike of one foot and next heel strike of the same foot), stride velocity (stride length divided by stride duration), normalized double-support time (time between heel strike of one foot to subsequent toe off of the opposite foot as a percentage of the time between left and right heel strikes), and step-length asymmetry using the following formula (19)

$$Asymmetry=100\times \left|\ln\left(\frac{left\;step\;length}{right\;step\;length}\right)\right|$$

where step length is the antero-posterior distance between feet at their respective heel strikes.

### Statistical Analysis

#### Single Participant Analysis

For each participant, tuning curves relating STN-DBS frequency (40–140 Hz) to each gait variable measured over multiple stride cycles from Door and No-Door trials separately were constructed. An ANOVA multiple comparison test with STN-DBS frequency as a factor was performed on each gait variable. This utilized Matlab's *multcompare* function with a Tukey's honest significant difference criterion in which non-overlapping confidence intervals indicated a significant difference (*P* < 0.05) between any pair of frequencies, thus identifying the best and non-separable STN-DBS frequencies for each gait variable for each patient. Better performance was indicated by higher values of stride velocity and length, and lower values of normalized double-support time and step-length asymmetry. Each patient's overall optimal STN-DBS frequencies were defined as the frequencies that were common to the best frequencies for each gait variable and both door conditions.

#### Group Analysis

These statistical tests were performed using SPSS (IBM SPSS ver.24). The Wilcoxon signed-rank test was used to compare the relative effectiveness of frequency vs. voltage, and to compare clinical postural scores at the time of the current test relative to post-surgery follow-up (measured off medication). Because identical STN-DBS frequencies were not always available when comparing scores at post-surgery (original frequency) with scores on the test day (test frequency) some corrections were adopted. If the original frequency was 130 Hz, the clinical score was compared with the mean measured at 140 and 120 Hz test frequencies during the experimental session. If the original frequency was above 140 Hz, the clinical score was compared with the one measured at 140 Hz test frequency.

The correlation between optimal frequency and clinical score was assessed using the non-parametric Spearman's rank correlation coefficient (r_s_). When a patient had more than one optimal frequency, the mean clinical score at those frequencies was paired with the mean optimal frequency.

Multiple regression with three factors (clinical score, right electrode site, left electrode site) as predictors of optimal frequency was performed using a forward stepwise method. Clinical scores were measured on the test day with the original stimulation frequency (as above). The left and right sites of the active electrode were determined as follows. The stereotactic coordinates of the center of each active contact were identified on an intra-operative stereotactic MRI scan (T2 weighted) acquired immediately following lead implantations. The coordinates were then superimposed onto the pre-implantation stereotactic T2-weighted MRI scan (20). From these images, the electrode contact locations were allocated to five regions of the STN (superior, postero-lateral, central, inferior, and antero-medial). Unipolar stimulation contacts in the superior and postero-lateral sites were merged to represent the dorsal region, and inferior and antero-medial sites were merged to represent ventral regions. An intermediate central region consisted solely of central sites. Bipolar stimulation sites were included in the analysis if both contacts were situated in the same merged region (left *n* = 2; right *n* = 2). If the two contacts were in different regions or if imaging was not available, they were designated as missing data (left *n* = 3; right *n* = 2). The regression data were subjected to automatic preparation, which trimmed outliers, replaced missing values and merged categories to maximize association with the target optimal frequency. The Akaike information criterion was used for entry/removal of a predictor from the model.

## Results

All patients tried to complete the tests at each of the six stimulation frequencies. However, patients and their carers were reminded that they could withdraw from a setting if it was too uncomfortable and not tolerable. Not all patients were able to tolerate the six stimulation frequencies, 40 Hz being the least well-tolerated. Six frequencies were completed by 11 patients, five by 8 patients, four by 4 patients, and three by 1 patient. However, this was deemed acceptable because we aimed to analyse each patient individually. If a frequency was not tolerated, clearly that frequency could not be considered as potentially optimal for that patient.

**Figure 1:**
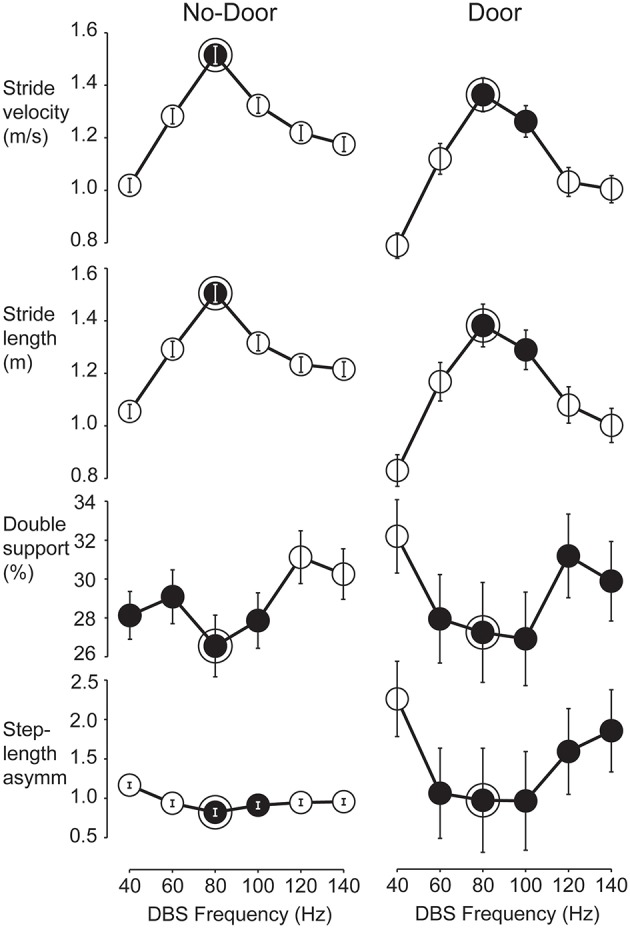
Tuning curves used to find the optimum STN-DBS frequency for gait in a representative patient. Mean values and confidence intervals used in the *multcompare* test from all strides performed by patient OP11 according to the six STN-DBS frequencies. Data shown separately for No-Door (left column) and Door (right column) conditions. Gait performance variables from top are stride velocity, stride length, normalized double-support and step-length asymmetry. Statistically, two frequencies were different when the plotted confidence interval did not overlap. For each variable, the statistical test was run and one or more best frequencies were identified (black circles). The optimal frequency band was defined as those best frequencies that were common to all gait variables in both the Door and No-Door condition (framed black circles). For this patient, the optimum frequency was 80 Hz.

The four variables chosen to represent gait performance were not equally sensitive to changes of stimulation frequency. Stride velocity was the most discriminatory variable (11 patients showed a single best frequency), followed by stride length (9 patients), double-support time (3 patients), and step-length asymmetry (2 patients). Reliance on just one gait variable was deemed insufficient to determine an optimal frequency per patient as it was important to ensure that a frequency that worked well for one gait variable was not detrimental to any others. We developed a common-frequency algorithm that included information from all four measured variables and their tuning curves under both Door and No-Door walking conditions (Figure 1).

**Figure 2:**
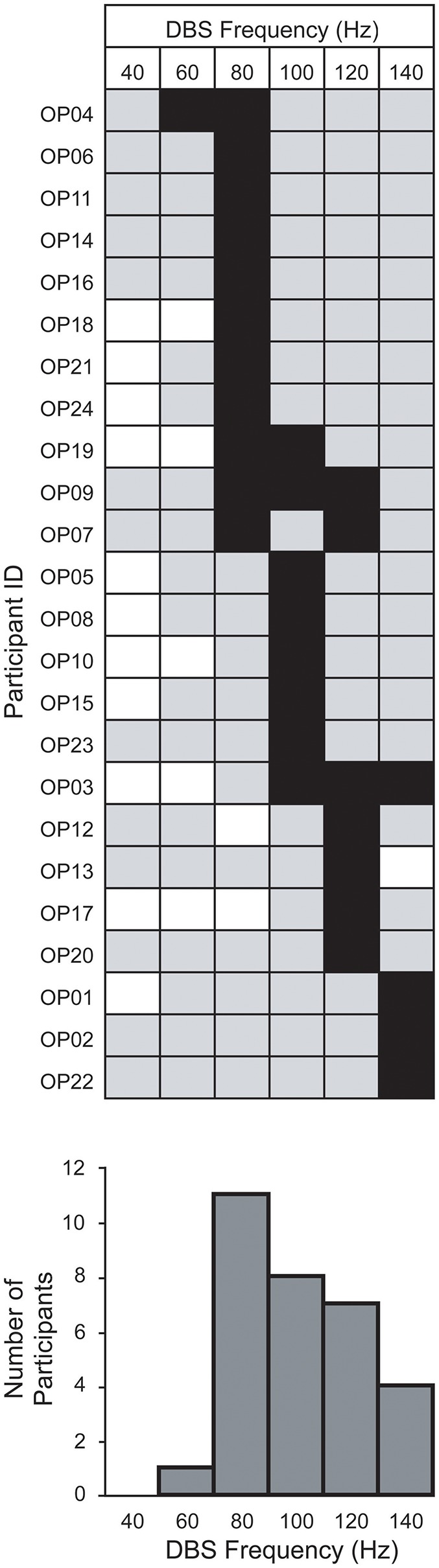
Within-group distribution of optimum STN-DBS frequencies for gait. The optimum frequencies (black squares) are shown among those studied (gray squares) and those not studied (white squares) for each patient, using the method described in Figure 1. Bar chart below indicates the number of patients for which a frequency was optimal.

Figure 2 shows the result of applying this common-frequency method to all patients individually. The method was able to identify a single optimal frequency for 19 patients. It was unable to distinguish between two frequencies in 3 patients and three frequencies in 2 patients. Every frequency studied, with the exception of 40 Hz, was designated as optimal for at least one patient (Figure 2). Of these, 60 Hz was the least represented frequency (*n* = 1) while 80 Hz was the most represented (*n* = 11).

### Frequency vs. Voltage

**Figure 3:**
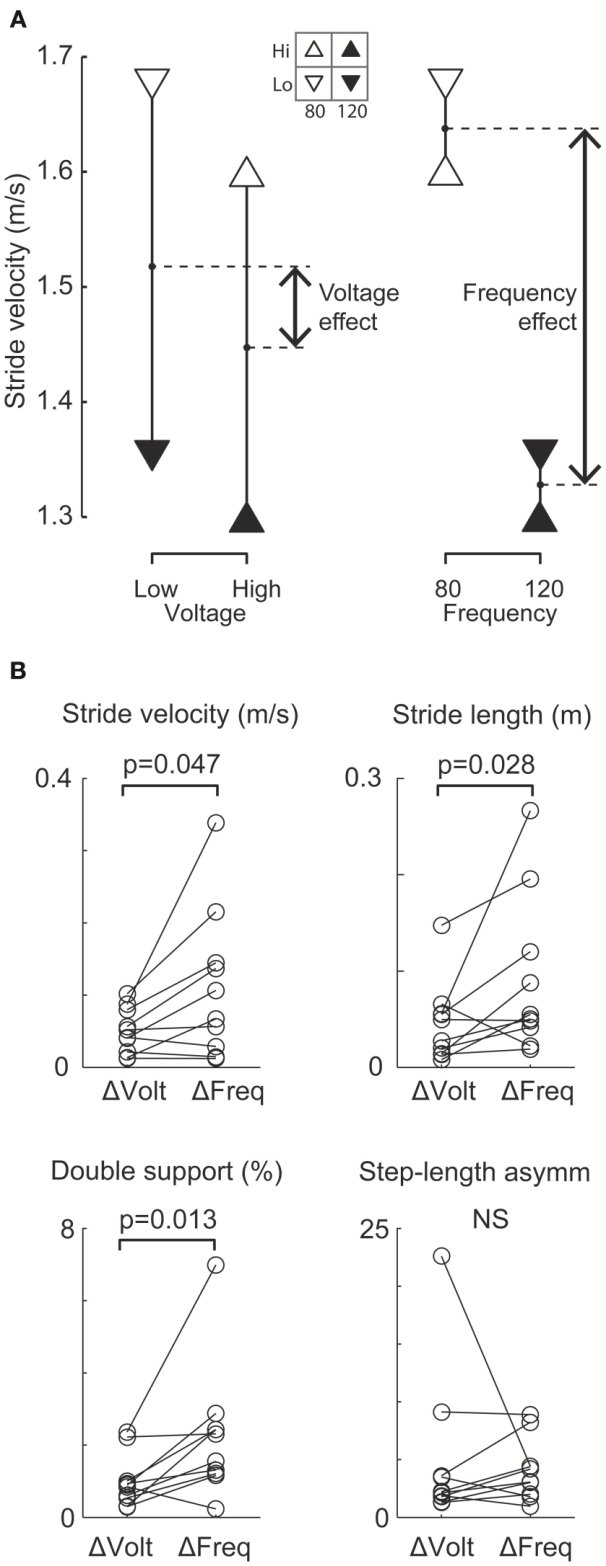
Comparison of relative effects of changing frequency and voltage on gait variables. **(A)** Representative patient's mean stride velocity data at each of four STN-DBS settings. The same data are arranged differently to show the mean effect of changing voltage (left) and the mean effect of changing frequency (right). For this patient, the change in stride velocity was larger when changing frequency from 120 to 80 Hz compared to a change in voltage from a high (4.3 and 5.2 V left and right electrodes respectively) to a low value (3.5 and 4.2V left and right electrode respectively). **(B)** All patients' frequency (Δ_Freq_) and voltage (Δ_Volt_) effects for each gait variable. The change due to Δ_Freq_ was significantly (*p* < 0.05) larger than the change due to Δ_Volt_ for stride velocity, stride length and double-support time.

To investigate whether stimulation frequency or voltage was more important for altering gait performance, a sub-set of patients were studied under four stimulation conditions using 2 frequencies (80 and 120 Hz) and two voltages (high and low). This reduced protocol was adopted in order to test the relative effect of stimulation frequency and voltage within the same patients. Although our results are based on ten patients and four conditions only, we observed that the four data points obtained for each variable from the four conditions can be arranged differently to show either the mean effect of voltage or the mean effect of frequency, as shown for one patient (Figure 3A). For the group (Figure 3B), the mean effect of frequency was reliably larger than the mean effect of voltage for 3 of the 4 gait variables (Wilcoxon: stride velocity, *P* = 0.047; stride length, *P* = 0.028; double-support time, *P* = 0.013; step-length asymmetry, *P* = 0.445).

### Axial Symptoms

The severity of gait and postural symptoms and their deterioration over the period of implantation was estimated from the reduced UPDRS score shown in Table 2. For the group, there was a trend for deterioration in clinical scores when comparing the patient post-surgery (original) and at test day with frequency set as close as possible to original (Wilcoxon between columns A and J in Table 2, *P* = 0.060). At the time of the current test session (i.e., Experiment 1), comparing scores measured at the best gait frequency (from columns C-H at frequency in I) and the scores measured at the frequency set at post-surgery follow-up (column J, from columns C-H in bold, i.e., frequency in B), the gait and postural symptoms were significantly better when stimulating at the best gait frequency identified in this study compared to the original frequency (Wilcoxon, *P* = 0.010). The current severity of gait and postural symptoms (at original frequency, column J) was inversely correlated with the optimal frequency for gait (column I, r_s_ = −0.410, *P* = 0.047). Over the period of implantation, the rate of change of clinical score (at original frequency, column L) also was inversely correlated with the optimal stimulation frequency (r_s_ = −0.417, *P* = 0.043). Thus, those patients with more severe and/or faster deteriorating symptoms, measured with the clinical score, tended to optimize at a lower stimulation frequency at the time of testing.

**Table 2:** Axial scores after surgery and at time of test with different DBS frequencies.

	**Post-surgery followup**		**Test day-clinical scores at DBS test frequencies**						**Test day**			
**column ID**	**(A)**	**(B)**	**(C)**	**(D)**	**(E)**	**(F)**	**(G)**	**(H)**	**(I)**	**(J)**	**(K)**	**(L)**
**patient ID**	Clinical score^*^	**DBS original freq (Hz)**	**40 Hz**	**60 Hz**	**80 Hz**	**100 Hz**	**120 Hz**	**140 Hz**	**Mean optimal gait freq (Hz)**	**Clinical score (column** ***C*** **to** ***H*****) at original freq [i.e.**, ***B*****]**^‡^	**Time since surgery (y)**	**Rate of clinical score change at original freq [i.e.**, ***(J-A)/K*****]**
OP01	1	130		10	8	4	**6**	4	140	5	10	0.44
OP02	2	130	5	6	3	5	**3**	3	140	3	5	0.25
OP03	2	130		4	4	3	3	**3**	120	3	9	0.17
OP04	3	130		4	3	5	**6**		70	6	8	0.43
OP05	2	130	2	3	2	2	**3**	**2**	100	2.5	4	0.17
OP06	2	130	5	3	2	2	**4**	**6**	80	5	6	0.55
OP07	1	160	3	3	2	2	1	**2**	100	2	13	0.17
OP08	5	160		8	4	8	8	**8**	100	8	6	0.60
OP09	6	130	4	3	3	3	**3**	**3**	100	3	7	−0.50
OP10	1	130			2	3	**3**	**2**	100	2.5	8	0.21
OP11	3	130	6	4	3	4	**5**	**5**	80	5	7	0.33
OP12	4	130	3	3		4	2	**4**	120	3	4	−0.33
OP13	1	130	3	2	2	2	2		120	2	7	0.17
OP14	3	130	5	1	2	1	**2**	**1**	80	1.5	5	−0.38
OP15	4	130	2	1	2	2	**2**	**2**	100	2	5	−0.50
OP16	0	130	2	3	5	3	**5**	**3**	80	4	10	0.44
OP17	4	200				2	0	**1**	120	1	8	−0.43
OP18	1	130			8	7	**9**	**8**	80	8.5	10	0.83
OP19	2	130			3	5	**6**	**5**	90	5.5	11	0.35
OP20	2	185	2	2	5	2	2	**2**	120	2	9	0.00
OP21	3	130		4	2	2	**2**	**2**	80	2	6	−0.20
OP22	4	130	4	3	6	2	**2**	2	140	2	4	−0.60
OP23	3	130	5	3	2	2	**4**	**3**	100	3.5	4	0.17
OP24	5	130		8	8	7	**8**	**10**	80	9	5	1.33

*Clinical scores measured off medication using items from UPDRS part III: Arise from chair, Posture, Gait, Pull test. Clinical scores collected at post-surgery follow-up are reported in column A, for the stimulation frequency chronically used at the time (column B). Clinical scores on test day at original frequency are reported in bold (column C-H, averaged in column J). Clinical scores on test day at optimal frequency are underlined (column C-H)*.

* *Recorded at 1 year after surgery except for OP03 (3 y), OP06 (0.5 y), OP07 (7 y), OP22 (0.67 y), OP24 (2 y). These differences were taken into account when calculating rate of axial score change*.

‡ *If patient's original frequency was not tested at test day, the score was taken as either the average of scores at the frequencies above and below original frequency or the score at the nearest frequency if original frequency was higher than test-day frequencies*.

### Site of Stimulation

**Figure 4:**
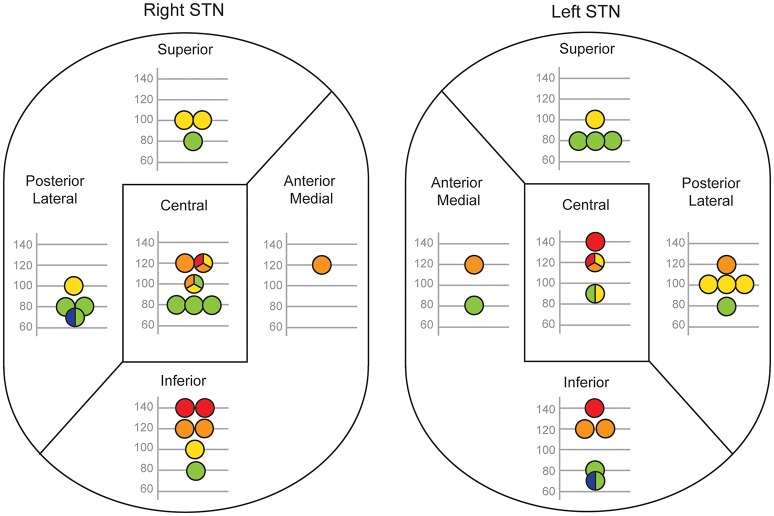
Relationship between electrode contact position and the optimal frequency for gait. Each patient's active contact location within five regions of STN is shown separately for their right and left electrodes. Only patients with monopolar stimulation are reported (right STN, *n* = 20; left STN, *n* = 19). The optimal frequency for gait is denoted by the position of the circle on the y-axis and by a color code (blue 60 Hz, green 80 Hz, yellow 100 Hz, orange 120 Hz, red 140 Hz). If more than one optimal STN-DBS frequency was found, the circle is split into two or three color-coded sections and its position on the y-axis is the mean of those frequencies.

Figure 4 shows each patient's optimum frequency for gait and their corresponding site of stimulation in the right and left STN divided into 5 regions. After merging these data into dorsal, central and ventral regions, and combining the right and left sides, a relationship between stimulation site and optimal frequency emerged. The median optimal stimulation frequency was significantly lower for the dorsal (90 Hz) than the ventral (120 Hz) sites (independent samples median test, *P* = 0.001). The median frequency in the central region (100 Hz) was intermediate.

### Dependence of Optimal Frequency on Axial Symptoms and Site of Stimulation

Because of the relationships between optimal frequency and both clinical score (current score at the original stimulation frequency) and stimulation site, we subjected the data to a multiple regression analysis. None of the predictors (clinical score, right, and left stimulation sites) were significantly correlated with each other. The regression model was significant [*F*_(2, 21)_ = 5.98, *P* = 0.009; adjusted *R*^2^ = 0.302; information criterion = 142.4] and retained the right STN site (coefficient = −20.034, *t* = −2.482, *P* = 0.022) and clinical score (coefficient = −3.540, *t* = −2.134, *P* = 0.045) as predictors, but rejected the left STN site. The model automatically merged the dorsal and central stimulation sites into a single region for the right STN predictor. Overall, the site of stimulation in the right STN had greater importance than the clinical score (0.575 vs. 0.425) as a predictor of optimal frequency.

## Discussion

In this study, we aimed to measure the effect of STN-DBS frequency on gait in finer detail than achieved previously. Our method was founded on measurements of gait variables over repeated stride cycles that reflect the performance of over-ground walking in PD. Stride length captured the small steps commonly observed in PD gait (21–23), stride velocity reflected bradykinesia, double-support time detected hesitations and freezing episodes (17), and step-length asymmetry captured the left-right asymmetry often observed in PD (19, 24). We explored a wide range of STN-DBS frequencies with the aim of finding the acute optimal stimulation frequency, in most cases with a resolution of 20 Hz, for each patient during withdrawal of their medication (≥12 h). Because of time constraints, this study did not additionally evaluate the effects of switching off the stimulator, but this did not detract from the aim of identifying the optimal frequency for a patient. The optimal frequency varied considerably between patients, but did not conform to the simple bimodal frequency distribution (high vs. low frequency) often implied in the literature (11–13, 16, 25–27). Instead, optimal frequencies were distributed continuously from 60 to 140 Hz. The principle used for determining an optimal frequency was that it should be no worse than any other frequency for all gait variables. This ensured that a frequency was not chosen if it improved one aspect of gait, but produced a decrement in performance for another aspect. Furthermore, this had to be the case when walking either without obstruction or when passing through a narrow doorway, a condition that has been shown to evoke gait difficulties in PD (17, 18). Satisfaction of these stringent criteria resulted in convergence on a single optimal frequency for around 80% of the patients. It is possible that a frequency resolution even finer than 20 Hz could have been established with this algorithm. However, the fact that we were unable to distinguish between two or three frequencies in 5 patients implied that we were close to the limit of practical frequency resolution.

### Is Frequency or Voltage the Important Stimulation Parameter?

Gait performance is described here as a function of STN-DBS frequency. However, a change in frequency was always accompanied by a change in stimulation voltage, in order to satisfy the criterion of constant energy stimulation. Was gait performance being altered by changes in voltage or frequency? To answer this definitively it would be necessary to map gait changes across a large number of frequency-voltage combinations. This would not be practically achievable in one session if sufficient time were allowed to elapse after a change in DBS settings (30 min in the current study) to achieve a stable behavioral effect (28). Our reduced version of this procedure showed that for four frequency-voltage combinations, frequency change affected gait performance more than voltage change. This suggests that frequency was likely to have been the more critical parameter across the range of settings that were employed in the study.

### What Are the Sources of Optimal-Frequency Variation?

The patient's clinical profile is one possible source of variation of optimal frequency. Previous studies have described the emergence of axial symptoms in chronically stimulated PD patients and how these symptoms are often not well-controlled by the typical high stimulation frequencies employed initially after surgery (11–14). We therefore investigated whether there was a relationship between the severity of axial symptoms measured clinically, or their rate of emergence, and the optimum frequency for gait. For both measures we found a significant negative correlation indicating that those patients with more severe and/or with a faster rate of emergence of axial symptoms tended to optimize at a lower frequency.

Khoo and colleagues showed a relationship between electrode contact within the STN and the preferred frequency of stimulation (11). Using a criterion of the best UPDRS-III score, they found that in 36% of cases, a patient's optimum electrode contact for 60 Hz stimulation in the STN was located more ventrally than his or her optimum contact for 130 Hz stimulation. This raised the possibility that the different frequency-gait tuning curves between our patients could have been related to differences in the location of the electrode contact within the STN. We did indeed find a relationship between the dorsal-to-ventral active contact location and optimal frequency, but our results are at odds with those of Khoo and colleagues (11). We found that dorsal sites were associated with low frequencies (median 90 Hz) and ventral sites with high frequencies (median 120 Hz). Our data, however, are consistent with the findings obtained by Xie and colleagues when they investigated the effect of STN stimulation through dorsal contacts in two patients with severe freezing of gait (27). They found that 60 Hz stimulation improved the patients' freezing of gait whereas 130 Hz stimulation made it worse.

The two key factors of stimulation site and severity of clinically assessed postural symptoms were unrelated. This is understandable given that electrode contact site was determined shortly after implantation based on effectiveness of improving overall clinical state, whereas the score considered here is just one sub-component of clinical state that was measured 4 or more years later at the time of testing. Nonetheless, both turned out to be significant predictors of the optimal frequency for gait when subjected to a multiple regression analysis. However, we found that the right STN stimulation site was a more important predictor of optimal frequency than the severity of symptoms. It is not clear why only the electrode site in the right STN was retained in the regression model; we were unable to find any conclusive evidence relating it to asymmetrical severity of symptoms.

### Why Should Optimal Frequencies Differ Between Patients?

One speculation for between-patient variation in optimal frequency is that a contribution to axial symptoms arises from the development of additional pathology outside the basal ganglia. A candidate is the pedunculopontine nucleus (PPN), which suffers significant cholinergic cell loss in PD (29). Experimental lesions of cholinergic PPN neurones in monkeys result in gait changes characterized by decreased step length and speed (30). Moreover, a PET study on imagined gait in PD suggested that STN-DBS may improve parkinsonian gait by modulating brainstem locomotor centers including the PPN (31). Presumably, additional PPN pathology would produce a greater gait disturbance in PD than pathology restricted to the basal ganglia. DBS applied directly to the PPN has been shown to improve motor function in some of the small number of patients in which it has been attempted, but the therapeutic frequency is generally less than that used for STN-DBS, ranging from 10 to 70 Hz (32–36). The STN connects with the PPN as well as the other basal ganglia nuclei in man (37), and so would be capable of exerting influence on both putative lesioned circuits. However, the STN-DBS frequency required may differ for the two circuits with a low frequency for the PPN and a high frequency for the basal ganglia. The optimal frequency for gait may be a compromise between a high and low frequency depending on the relative disruption of function caused by each of the two lesions. Such a mechanism could explain why the optimal frequency for gait was continuously distributed between 60 and 140 Hz in our cohort. It also provides an explanation for why patients with greater axial symptoms, arguably implying greater PPN disruption, tend to optimize at a lower frequency. However, it is not clear whether this model can also explain why active electrode contacts located in the dorsal STN should favor lower frequencies compared to ventral contacts. One possibility could be that STN projections to PPN are not uniformly distributed throughout the STN, but have stronger connections in more dorsal regions. More detailed neuro-anatomy of these regions in man than is currently available would be required to investigate this hypothesis.

### Study Limitations

In this study, we chose to use a reduced version of the UPDRS-III test as a clinical assessment of axial symptoms. Other clinical tests may be considered more comprehensive in their analysis of the different postural domains affected, but our choice of test was limited by the time constraints of the demanding protocol and to enable comparisons with prior clinical assessments of axial function in the same patients. The rater for the clinical assessment of axial function was an experienced clinician, but was not blinded to stimulation condition. Although this is a limitation, it was an inevitable consequence of the same clinician being responsible for setting the DBS parameters. The second experiment, which investigated the relative effect of stimulation voltage and frequency on gait performance, had limitations as it was undertaken and completed by only 42% of the group and four representative voltage-frequency conditions were mapped, out of all possible combinations. Finally, the quantitative assessment of DBS frequency on gait performance was performed over a short time-scale, i.e., started 30 min after a change in stimulation setting and completed within 30 min (60 min in total). Whether, the measured gait performance at a given frequency keeps stable over a longer time-scale remains an open question.

## Conclusion

In conclusion, the gait performance of PD patients receiving STN-DBS for four or more years was sensitive to relatively small changes in stimulation frequency. In 79% of patients it was possible to determine statistically an optimum frequency for each individual's gait performance with a resolution of 20 Hz. The optimum frequency varied considerably between patients and was associated with both the dorsal-ventral location of the electrode contact site and the severity of their axial symptoms. This variability may stem from variable progression of pathology outside the basal ganglia.

## Author Contributions

IDG: design of experiment, data collection, analysis and interpretation, article writing and revising. EK, DG, and AP: design of experiment, participant recruitment, data collection and analysis, article revising. DV: design of experiment, article revising. HA: data analysis, article revising. TF: participant recruitment, article revising. PL: conception and design of the experiment, participant recruitment, article revising. BD: conception and design of the experiment, data analysis and interpretation, article writing and revising.

### Conflict of Interest Statement

PL and TF have received honoraria for speaking at meetings sponsored by Medtronic, the device manufacturer for this study. The remaining authors declare that the research was conducted in the absence of any commercial or financial relationships that could be construed as a potential conflict of interest.

## Abbreviations

PD: Parkinson's disease
DBS: deep brain stimulation
PPN: pedunculopontine nucleus
STN: subthalamic nucleus
UPDRS: Unified Parkinson's disease rating scale.

## References

[1] Derost PP Ouchchane L Morand D Ulla M Llorca PM Barget M. Is DBS-STN appropriate to treat severe Parkinson disease in an elderly population? Neurology (2007) 68:1345–55. 10.1212/01.wnl.0000260059.77107.c2 17452578

[2] Krack P Batir A Van Blercom N Chabardes S Fraix V Ardouin C. Five-year follow-up of bilateral stimulation of the subthalamic nucleus in advanced Parkinson's disease. N Engl J Med. (2003) 349:1925–34. 10.1056/NEJMoa035275 14614167

[3] Liang GS Chou KL Baltuch GH Jaggi JL Loveland-Jones C Leng L. Long-term outcomes of bilateral subthalamic nucleus stimulation in patients with advanced Parkinson's disease. Stereotac Funct Neurosurg. (2006) 84:221–7. 10.1159/000096495 17063043

[4] Merola A Zibetti M Angrisano S Rizzi L Ricchi V Artusi CA Parkinson's disease progression at 30 years: a study of subthalamic deep brain-stimulation patients. Brain (2011) 134:2074–84. 10.1093/brain/awr121 21666262

[5] Ostergaard K Sunde N Dupont E. Effects of bilateral stimulation of the subthalamic nucleus in patients with severe Parkinson's disease and motor fluctuations. Mov Disord. (2002) 17:693–700. 10.1002/mds.10188 12210858

[6] Rodriguez-Oroz MC Obeso JA Lang AE Houeto JL Pollak P Rehncrona S. Bilateral deep brain stimulation in Parkinson's disease: a multicentre study with 4 years follow-up. Brain (2005) 128:2240–9. 10.1093/brain/awh571 15975946

[7] Schlenstedt C Shalash A Muthuraman M Falk D Witt K Deuschl G. Effect of high-frequency subthalamic neurostimulation on gait and freezing of gait in Parkinson's disease: a systermatic review and meta-analysis. Eur J Neurol. (2017) 24:18–26. 10.1111/ene.13167 27766724

[8] St. George RJ Nutt JG Burchiel KJ Horak FB. A meta-regression of the long-term effects of deep brain stimulation on balance and gait in PD. Neurology (2010) 75:1292–9. 10.1212/WNL.0b013e3181f61329 20921515 PMC3013496

[9] Tornqvist AL Schalen L Rehncrona S. Effects of different electrical parameter settings on the intelligibility of speech in patients with Parkinson's disease treated with subthalamic deep brain stimulation. Mov Disord. (2005) 20:416–23. 10.1002/mds.20348 15593314

[10] Tripoliti E Zrinzo L Martinez-Torres I Frost E Pinto S Foltynie T. Effects of subthalamic stimulation on speech of consecutive patients with Parkinson disease. Neurology (2011) 76:80–6. 10.1212/WNL.0b013e318203e7d0 21068426 PMC3262409

[11] Khoo HM Kishima H Hosomi K Maruo T Tani N Oshino S Low-frequency subthalamic nucleus stimulation in Parkinson's disease: a randomized clinical trial. Mov Disord. (2014) 29:270–4. 10.1002/mds.25810 24449169

[12] Moreau C Defebvre L Destee A Bleuse S Clement F Blatt JL. STN-DBS frequency effects on freezing of gait in advanced Parkinson disease. Neurology (2008) 71:80–4. 10.1212/01.wnl.0000303972.16279.46 18420482

[13] Ramdhani RA Patel A Swope D Kopell BH. Early use of 60 Hz frequency subthalamic stimulation in Parkinson's disease: a case series and review. Neuromodulation (2015) 18:664–9. 10.1111/ner.12288 25833008

[14] Xie T Vigil J MacCracken E Gasparaitis A Young J Kang W. Low-frequency stimulation of STN-DBS reduces aspiration and freezing of gait in patients with PD. Neurology (2015) 84:1–6. 10.1212/WNL.0000000000001184 25540305 PMC4336001

[15] Sidiropoulos C Walsh R Meaney C Poon YY Fallis M Moro E. Low-frequency subthalamic nucleus deep brain stimulation for axial symptoms in advanced Parkinson's disease. J Neurol. (2013) 260:2306–11. 10.1007/s00415-013-6983-2 23749331

[16] Vallabhajosula S Haq IU Hwynn N Oyama G Okun M Tillman MD. Low-frequency versus high-frequency subthalamic nucleus deep brain stimulation on postural control and gait it Parkinson's disease: a quantitative study. Brain Stimul. (2015) 8:64–75. 10.1016/j.brs.2014.10.011 25440578

[17] Cowie D Limousin P Peters A Hariz M Day BL. Doorway-provoked freezing of gait in Parkinson's disease. Mov Disord. (2012) 27:492–9. 10.1002/mds.23990 21997389

[18] Cowie D Limousin P Peters A Day BL. Insights into the neural control of locomotion from walking through doorways in Parkinson's disease. Neuropsychologia (2010) 48:2750–7. 10.1016/j.neuropsychologia.2010.05.022 20519135

[19] Yogev G Plotnik M Peretz C Giladi N Hausdorff JM. Gait asymmetry in patients with Parkinson's disease and elderly fallers: when does the bilateral coordination of gait require attention? Exp Brain Res. (2007) 177:336–46. 10.1007/s00221-006-0676-3 16972073

[20] Foltynie T Zrinzo L Martinez-Torres I Tripoliti E Petersen E Holl E. MRI-guided STN DBS in Parkinson's disease without microelectrode recording: efficacy and safety. J Neurol Neurosurg Psychiatry (2011) 82:358–63. 10.1136/jnnp.2010.205542 20571041

[21] Griffin HJ Greenlaw R Limousin P Bhatia K Quinn NP Jahanshahi M. The effect of real and virtual cues on walking in Parkinson's disease. J Neurol. (2011) 258:991–1000. 10.1007/s00415-010-5866-z 21221626

[22] Okada Y Fukumoto T Takatori K Nagino K Hiraoka K. Abnormalities of the first three steps of gait initiation in patients with Parkinson's disease with freezing of gait. Parkinsons Dis. (2011) 2011:202937. 10.4061/2011/202937 21785691 PMC3140034

[23] Rocchi L Chiari L Mancini M Carlson-Kuhta P Gross A Horak FB. Step initiation in Parkinson's disease: influence of initial stance conditions. Neurosci Lett. (2006) 406:128–32. 10.1016/j.neulet.2006.07.027 16901637

[24] Lee CS Schulzer M Mak E Hammerstad JP Calne S Calne DB Patterns of asymmetry do not change over the course of idiopathic parkinsonism: implications for pathogenesis. Neurology (1995) 45(3 Pt1):435–9. 10.1212/WNL.45.3.435 7898691

[25] Ricchi V Zibetti M Angrisano S Merola A Arduino N Artusi. Transient effects of 80 Hz stimulation on gait in STN DBS treated PD patients: a 15 months follow-up study. Brain Stimul. (2012) 5:388–92. 10.1016/j.brs.2011.07.001 21824834

[26] Timmermann L Wojtecki L Gross J Lehrke R Voges J Maarouf M. Ten-hertz stimulation of subthalamic nucleus deteriorates motor symptoms in Parkinson's disease. Mov Disord. (2004) 19:1328–33. 10.1002/mds.20198 15389990

[27] Xie T Kang UJ Warnke P. Effect of stimulation frequency on immediate freezing of gait in newly activated STN DBS in Parkinson's disease. J Neurol Neurosurg Psychiatry (2012) 83:1015–7. 10.1136/jnnp-2011-302091 22696586

[28] Moro E Esselink RJA Xie J Hommel M Benabid AL Pollak P. The impact on Parkinson's disease of electrical parameters settings in STN stimulation. Neurology (2002) 59:706–13. 10.1212/WNL.59.5.706 12221161

[29] Hirsch E Graybiel AM Duyckaerts C Javoy-Agid F. Neuronal loss in the pedunculopontine tegmental nucleus in Parkinson disease and in progressive supranuclear palsy. Proc Natl Acad Sci USA. (1987) 84:5976–80. 10.1073/pnas.84.16.5976 3475716 PMC298986

[30] Karachi C Grabli D Bernard FA Tande' D Wattiez N Belaid H. Cholinergic mesencephalic neurons are involved in gait and postural disorders in Parkinson disease. J Clin Invest. (2010) 120:2745–54. 10.1172/JCI42642 20628197 PMC2912198

[31] Weiss PH Herzog J Pötter-Nerger M Falk D Herzog H Deuschl G. Subthalamic nucleus stimulation improves parkinsonian gait via brainstem locomotor centers. Mov Disord. (2015) 30:1121–5. 10.1002/mds.26229 25914247

[32] Ferraye MU Debû B Fraix V Goetz L Ardouin C Yelnik J. Effects of pedunculopontine nucleus area stimulation on gait disorders in Parkinson's disease. Brain (2010) 133:205–14. 10.1093/brain/awp229 19773356

[33] Mazzone P Lozano A Stanzione P Galati S Scarnati E Peppe A Implantation of human pedunculopontine nucleus: a safe and clinically relevant target in Parkinson's disease. Neuroreport (2005) 26:1877–81. 10.1097/01.wnr.0000187629.38010.12 16272871

[34] Moro E Hamani C Poon Y-Y Al-Khairallah T Dostrovsky JO Hutchinson WD. Unilateral pedunculopontine stimulation improves falls in Parkinson's disease. Brain (2010) 133:215–24. 10.1093/brain/awp261 19846583

[35] Plaha P Gill SS. Bilateral deep brain stimulation of the pedunculopontine nucleus in Parkinson's disease. Neuroreport (2005) 16:1883–7. 10.1097/01.wnr.0000187637.20771.a0 16272872

[36] Stefani A Lozano AM Peppe A Stanzione P Galati S Tropepi D. Bilateral deep brain stimulation of the pedunculopontine and subthalamic nuclei in severe Parkinson's disease. Brain (2007) 130(Pt 6):1596–607. 10.1093/brain/awl346 17251240

[37] Aravamuthan BR Muthusamy KA Stein JF Aziz JF Johansen-Berg H. Topography of cortical and subcortical connections of the human pedunculopontine and subthalamic nuclei. Neuroimage (2007) 37:694–705. 10.1016/j.neuroimage.2007.05.050 17644361

